# High Hepsin expression predicts poor prognosis in Gastric Cancer

**DOI:** 10.1038/srep36902

**Published:** 2016-11-14

**Authors:** Mingming Zhang, Junjie Zhao, Wenyi Tang, Yanru Wang, Peike Peng, Lili Li, Shushu Song, Hao Wu, Can Li, Caiting Yang, Xuefei Wang, Chunyi Zhang, Jianxin Gu

**Affiliations:** 1 Department of Biochemistry and Molecular Biology, School of Basic Medical Sciences, Fudan University, Shanghai 200032, China; 2 Department of General Surgery, Zhongshan Hospital, Fudan University, Shanghai 200032, China; 3 Department of Anatomy and Histology & Embryology, School of Basic Medical Sciences, Fudan University, Shanghai 200032, China

## Abstract

Hepsin, a membrane-associated serine protease, is frequently upregulated in epithelial cancers and involved in cancer progression. Our study aims to describe the expression pattern and evaluate the clinical implication of hepsin in gastric cancer patients. The mRNA expression of hepsin was analyzed in 50 gastric cancer and matched non-tumor tissues, which was downregulated in 78% (39/50) of gastric cancer. By searching and analyzing four independent datasets from Oncomine, we obtained the similar results. Furthermore, we evaluated the hepsin expression by IHC in tissue microarray (TMA) containing 220 Gastric Cancer specimens. More importantly, Kaplan-Meier survival and Cox regression analyses were taken to access the prognosis of gastric cancer and predicted that hepsin protein expression was one of the significant and independent prognostic factors for overall survival of Gastric Cancer.

Gastric cancer, one of the most common malignant cancers in the world, is responsible for the second leading cause of cancer death in both sexes worldwide, although the incidence has decreased over past several decades^1^,^2^. And most patients diagnosed with gastric cancer at advanced stage have a low 5-year survival rate^3^. The crucial reason for the poor prognosis of the most malignant cancers including gastric cancer is invasion and metastasis^4^. Traditionally, the tumor-node-metastasis (TNM) classification of International Union Against Cancer is composed of tumor invasive depth, lymph node metastasis and distant metastasis. Because of the heterogeneity of the tumor, patients of gastric cancer in the same stage may have diverse outcomes^5^. To improve the life quality and survival rate of gastric cancer patients, some novel and effective therapeutic methods must be created and developed.

Serine proteases have been known to mediate many biological processes. Hepsin (also called TMPRSS1), a putative type II transmembrane serine protease (TTSPs) of 417 amino acid residues, was originally identified as a human liver cDNA clone^6^. Hepsin has the active site triad residues of His(H), Asp(D), and Ser(S), which make up the catalytic triad common to all serine proteases and participate in enzyme catalysis^7^,^8^. The mRNA for hepsin is present in many tissues, with the high level in the liver tissue, and the low level in the kidney, pancreas, lung, thyroid, pituitary gland, and the testis^9^. Furthermore, hepsin is frequently overexpressed in prostate cancer^10^,^11^,^12^,^13^, breast cancer^14^, ovarian cancer^15^,^16^ and renal cell carcinoma^17^ and implicated in augmenting the prostate and ovarian tumorigenesis and metastatic dissemination^16^,^18^,^19^,^20^, mammalian cell growth and maintenance of cell of morphology^21^, activation of human Factor VII, blood coagulation and thrombin formation^22^ and developmental processes, such as blastocyst hatching^23^. Since hepsin is a transmembrane serine protease, it may play significant roles in biological and pathological processes occurring on the cell surfaces, including participating in signal transduction, processing of protein hormones, growth factors and receptors and activation of latent proteases. The expression pattern and impact of hepsin in gastric cancer remain unclear, so we aim to investigate the correlation between hepsin protein and overall survival (OS) of gastric cancer patients.

In this study, we examined the hepsin expression pattern in gastric cancer and its relationship with clinicopathological characteristics. Moreover, we generated a predictive nomogram to delineate the 3- and 5-year overall survival of the patients with gastric cancer after surgery.

## Results

### Hepsin mRNA and protein expression in Gastric cancer

**Figure 1:**
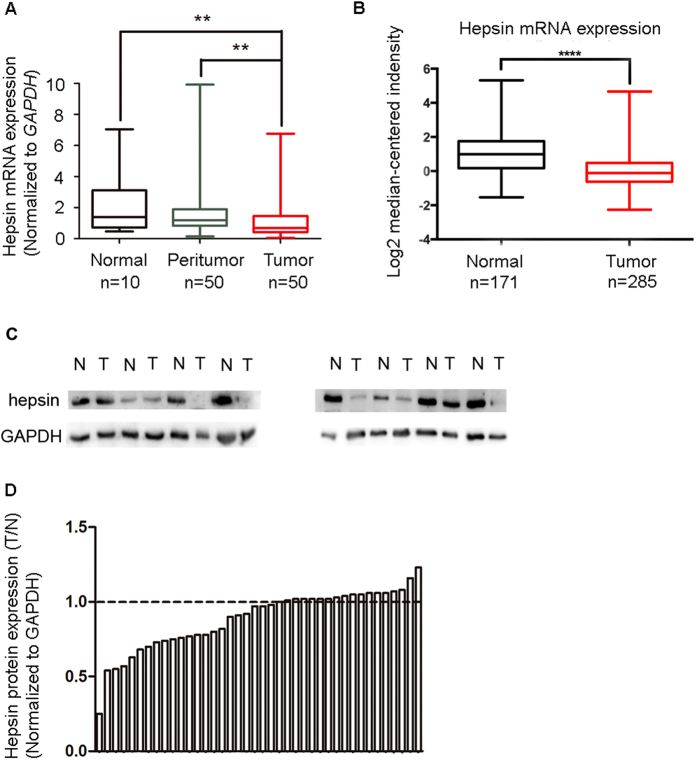
Hepsin expression is decreased in gastric cancer at mRNA and proetein level. (**A**) The Hepsin mRNA expression levels in 10 normal tissues, 50 matched peritumor and tumor tissues were determined by real-time quantitative PCR. (**B**) The Hepsin mRNA expression levels in five independent microarray datasets from Oncomine database reported by Cho, Cui, Wang, Chen, and DErrico. (**C**) Western blotting analysis of hepsin expression in gastric cancer 40 pairs of resection specimens from gastric cancer patients. (P: peritumor, T: tumor) (**D**) The relative hepsin protein expression levels in 40 pairs of representative gastric cancer tumor tissues and the matched adjacent non-tumor tissues.

**Figure 2:**
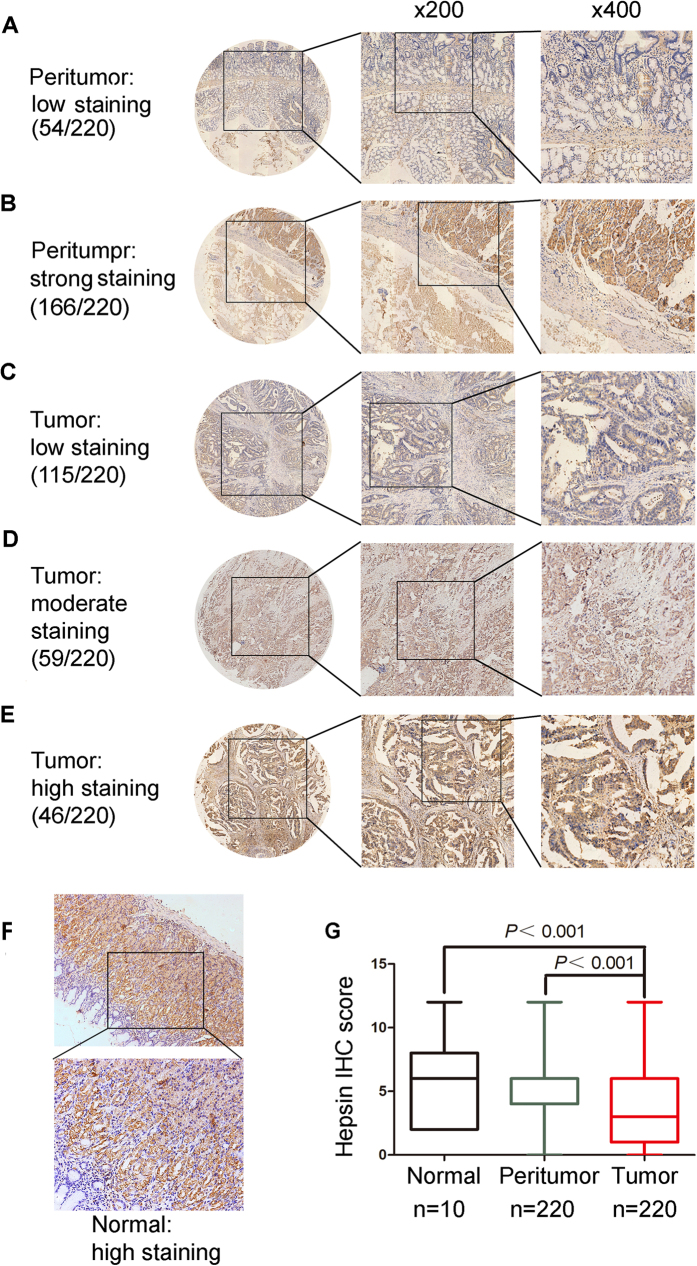
Hepsin expression in gastric cancer tissue samples. (**A–F**) Representative images of hepsin protein expression in gastric cancer tissues, compared with corresponding peritumor tissues and normal tissues; (**A**) peritumor tissue, scored as low. (**B**) Peritumor tissue, scored as strong. (**C**) Gastric cancer tissue, scored as low; (**D**) gastric cancer tissue, scored as moderate; (**E**) gastric cancer tissue, scored as high; (**F**) normal tissue, scored as high; (**G**) Comparison of hepsin IHC density between normal gastric tissues and gastric cancer tissues.

We used real-time quantitative PCR to determine Hepsin mRNA expression level in 10 normal tissues, 50 gastric cancer tumor tissues and patient-matched adjacent peritumor tissues. Representative images of three tumor samples stained by hematoxylin-eosin (HE) (Supplemental Figure 2). The tumor samples have similar tumor cell content (60% approximately) would be used to detect the mRNA or protein expression according to HE staining. The Hepsin mRNA expression level was downregulated in 78% (39/50) of gastric cancer patients (Fig. 1A). Furthermore, we searched and analyzed Hepsin mRNA expression in five independent microarray datasets from Oncomine database (Cho, Cui, Wang, Chen, and DErrico) (Fig. 1B, Supplemental Figure 1), which is consistent with our data. Hepsin protein expression levels were examined by western blot and immunohistochemical staining (IHC) methods (Figs 1C and 2). As shown in Fig. 1C, one protein bands, whose molecular weight is about 42 kDa, is observed in gastric cancer tissues and matched adjacent pericancer tissues in western blot analysis. A decrease in hepsin expression was observed in 55% (22/40) of the gastric cancer tumor tissues compared with the matched adjacent peritumor tissues (Fig. 1C,D). Further, the expression of hepsin protein was examined in a tissue microarray (TMA) containing 220 pairs of gastric tissues by IHC staining analysis. Evidence was presented that hepsin protein expression was mainly located in the nuclear and cytoplasm of gastric tumor cells, peritumoral tissue cells and normal tissue cells (Fig. 2). The IHC density of hepsin exhibits a significant difference in gastric tumor tissues and their matched adjacent non-tumor tissues (P < 0.001) (Fig. 2G).

Hepsin is a transmembrane serine protease and has one predicted N-glycosylation site in Asn-112. We found that N-glycosylation at Asn-112 is important for hepsin cell surface targeting and tumor invasion and migration. Wild hepsin was mainly localized in the cytoplasm and the cell membrane, while hepsin mutant N112Q was localized predominantly in the nucleus of the AGS cells (Supplemental Figure 3). So we speculated that nuclear hepsin in IHC should be de-glycosylated or not fully glycosylated. In addition, wild hepsin overexpression enhanced cell migration and invasion (Supplemental Figure 4), whereas hepsin mutant N112Q attenuated the invasion and metastatic potential compared to wild hepsin in MGC80-3 cells (Supplemental Figure 4). Similar roles of N-glycosylation in regulating cell surface expression and protease activity have been reported in other type II transmembrane serine proteases, such as corin^24^, enteropeptidase^25^, matriptase^26^, and matriptase-2^27^, which are involved in blood pressure regulation, food digestion, epithelial function, and iron metabolism, respectively^24^,^28^,^29^.

### Relationship between Hepsin expression and clinical parameters

To determine the clinical significance of hepsin expression in gastric cancer, the Chi-square test was taken to assess the associations between hepsin protein expression and clinicopathological parameters (including age, gender, tumor location, Histological differentiation, Lauren classification, T classification, N classification, distant metastasis, clinical stage and intravascular cancer emboli). The results demonstrated that hepsin expression in gastric cancer tissues is closely associated with histological differentiation (P = 0.001), Lauren classification (P = 0.001), T classification (P = 0.011) N classification (P = 0.039) and clinical stage (P = 0.007). No significant associations were detected between hepsin expression and age, gender, tumor location, distant metastasis or intravascular cancer emboli (Table 1). The results were confirmed in the validation set of patients. It was also demonstrated that hepsin expression was correlated to Lauren classification (P = 0.038), T classification (P = 0.033) N classification (P = 0.031) and clinical stage (P = 0.023) (Supplementary Table 1).

### Correlation between Hepsin expression and prognosis in Gastric cancer patients

**Figure 3:**
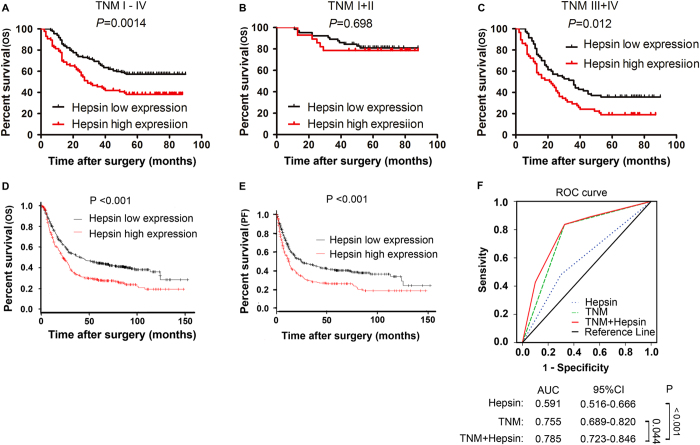
Hepsin expression is correlated with the overall survival rate in gastric cancer patients. (**A**) Kaplan-Merier survival curves show high expression level of hepsin was significantly correlated with poor survival of gastric cancer. (**B,C**) Comparisons of overall survival between hepsin high expression and hepsin low expression in early clinical stage (I–II) cohort and in advanced clinical stage (III–IV) cohort. (**D**,**E**) The prognostic value of Hepsin in gastric cancer was verified by online survival analysis software (http://www.kmplot.com/analysis/index.php?p=service&cancer=gastric), which integrated reported microarray datasets. It also showed that high expression of Hepsin was significantly associated with poorer overall survival as well as progression-free survival in gastric cancer patients (P < 0.001). (**F**) ROC analysis of the sensitivity and specificity for the prognosis of overall survival by TNM stage/hepsin expression model, TNM stage model, and hepsin expression model. P-values were calculated by log-rank test.

To further evaluate the prognostic value of hepsin in gastric cancer, we explored the correlation between hepsin expression and clinical data by Kaplan-Meier analysis and log-rank test. As shown in Fig. 3, high hepsin expression was associated with poor overall survival (P = 0.0014). Furthermore, we explored the association between hepsin expression and overall survival in gastric cancer patients with early or advanced clinical stages and with or without lymphatic metastasis and vascular invasion. According to Kaplan-Meier analyses, we found the overall survival is shorter in gastric cancer patients with high hepsin expression in all stages (Fig. 3A) or in III–IV stage (Fig. 3C). Similar result was confirmed in our validation set (Supplementary Figure 5). High expression of hepsin was also found to be associated with poor overall survival in gastric cancer patients without vascular invasion or with lymphatic metastasis (Supplemental Figure 6). Consistent with our results, the prognostic value of hepsin in gastric cancer was verified by online survival analysis software (http://www.kmplot.com/analysis/index.php?p=service&cancer=gastric), which integrated reported microarray datasets. The result demonstrated that high expression of hepsin correlated to poorer overall survival (Fig. 3D) as well as progression-free survival (Fig. 3E) in gastric cancer patients. In order to obtain a more sensitive predictive model for outcomes of gastric cancer patients, we combined hepsin expression and TNM stage to create a prognostic score system. ROC analysis revealed that the combination of hepsin and TNM stage showed better prognostic value [area under curve (AUC) 0.785, 95% confidence interval (CI) 0.723–0.846] than TNM stage alone (AUC 0.755, 95% CI 0.689–0.820, P = 0.044) or hepsin expression alone (AUC 0.591, 95% CI 0.516–0.666, P < 0.001) (Fig. 3F). We also analyzed the recurrence free survival information of GSE26253 datebase (Supplemental Figure 7).

In addition, univariate and multivariate analyses showed that hepsin could be useful as an independent risk factor for poor prognosis in the 220 cases of gastric cancer. The univariate Cox regression analyses showed that T classification (P < 0.001), N classification (P < 0.001), distant metastasis (P < 0.001), clinical TNM stage (P < 0.001), Intravascular cancer emboli (P = 0.0024) and hepsin expression (P = 0.0014) were significantly relevant with overall survival in gastric cancer. The multivariate Cox regression analyses, however, showed that T classification (P = 0.016), distant metastasis (P < 0.001), clinical TNM stage (P = 0.011) and hepsin expression (P = 0.036) were significantly different (Table 2). Then the Harrell’s concordance index (C-index) analyses were examined to assess the predictive accuracies of TNM stage and hepsin protein expression (Table 3).

### Nomogram and calibration plot analyses for gastric cancer patients

**Figure 4:**
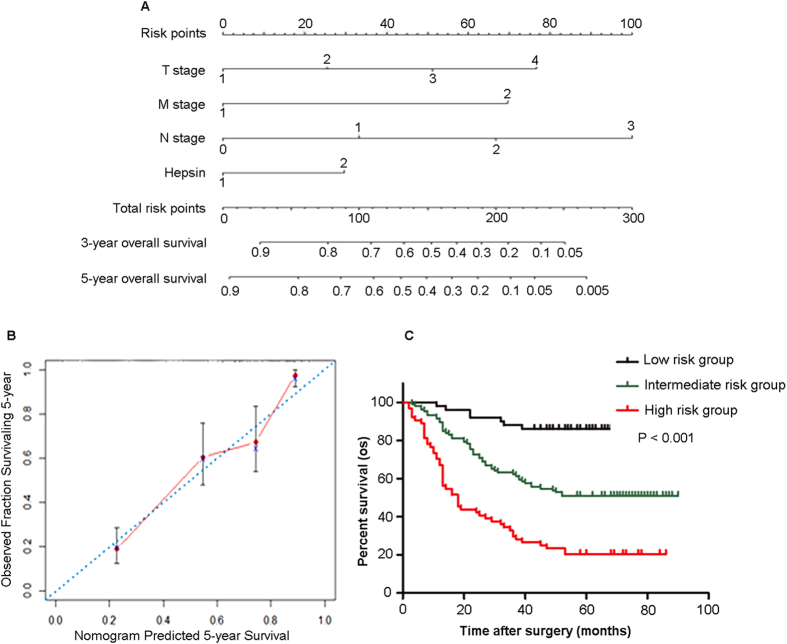
Nomogram and calibration plot analyses for the predictive value of hepsin protein expression in gastric cancer patients. (**A**) Nomogram generation for predicting overall survival integrated with T stage (1 represents T1, 2 represents T2, 3 represents T3, 4 represents T4), N stage (0 represents N0, 1 represents N1, 2 represents N2, 3 represents N3), distant metastasis (0 represents absence, 1 represents presence), and hepsin expression (0 represents low expression, 1 represents high expression). (**B**) Calibration curve for nomogram-predicted and observed 5-year overall survival. (**C**) The patients were stratified into 3 groups according to their percentile of the nomogram-predicted score: low risk group (<25th percentile), intermediate risk group (25th–75th percentile), and high risk group (>75th percentile).

Based on obtained evidence, we used patients’ data in the two cohorts to develop a nomogram to predict OS at 3 and 5 years after surgery (Fig. 4A). The predictors included tumor T stage, N stage, M stage and hepsin expression, all of which were independent prognostic indicators for OS. In the nomogram, a higher total point represents a worse survival. The calibration plot predicted 5-year overall survival were built to give the internal validation, which performed well compared with the ideal model (Fig. 4B). We next stratified the gastric cancer patients into 3 groups according to the score calculated using the nomogram: low-risk (<25th percentile), intermediate-risk (25th–75th percentile), and high-risk (>75th percentile) groups (Fig. 4C). As shown in Fig. 4C, the nomogram could effectively discriminate the risk of OS in gastric cancer patients.

## Discussion

Although gastric cancer incidence has declined for decades, it remains the fifth most common cancer and the fifth leading cause of cancer-related mortality in USA^30^. The majority of gastric cancer patients are diagnosed at advanced stage, due to lacking early detecting methods^31^. To date, the outcomes for gastric cancer patients with similar TNM stage can be very different because of the heterogeneity of this tumor, and the prognostic models for gastric cancer patients are largely relied on the TNM stage^5^. Therefore, we need to identify some novel molecules associated with tumorigenesis of gastric cancer and better understand the tumor progression and predict the cancer clinical outcomes. It will be helpful to compare expression levels of mRNA and protein of hepsin in tumor tissues with matched normal tissues of gastric cancer patients to describe the physiological and pathophysiological importance of hepsin in gastric cancer.

To this end, we have first time described the hepsin expression pattern in gastric cancer tissues in both protein and mRNA levels in this study. Additionally, the hepsin protein expression and its relationship with the clinicopathological parameters and clinical prognosis values are illustrated.

In the study, hepsin mRNA and protein expression level is mainly downregulated in Gastric cancer tissues. High expression of hepsin is correlated with poorer overall survival, as well as progression-free survival in patients with gastric cancer. There is a significance between high hepsin expression and low hepsin expression in gastric cancer patients with advanced stage, III–IV, so we speculate that hepsin may contribute to gastric cancer in later stage. In Zuyan Luo’s study, the OLFM4 expression pattern and correlation with gastric cancer patients’ overall survival are similar with our results^32^. Our findings reflect distinct actions of gastric and gastric carcinoma cells in response to hepsin protein. Probably, gastric and gastric carcinoma cells use different signaling pathways in response to hepsin expression. For example, hepatocytes react differently than hepatoma cells to IL-6 stimulation in regulating HBV replication. In hepatoma cells, IL-6 stimulates HBV transcription by activating STAT-3, which interacts with HNF3 bound to the HBV enhancer^33^. However, in primary human hepatocytes, IL-6 suppresses HBV gene expression and replication through the down-regulation of HNF4a and HNF1a^34^.

Although a large quantity of membrane-associated proteinase has been found, their biological roles are still unknown. Associations between TMPRSS2 and TMPRSS4 and cancers have been reported. TMPRSS2 may be a potential diagnostic or therapeutic target for prostate cancer, which is considered to have a role in cell biology^35^. TMPRSS4 is usually overexpressed in pancreatic cancer, however, its functional significance remains to be illustrated^36^. Low expression levels of hepsin and TMPRSS3 are associated with poor breast cancer survival^37^. Because hepsin is upregulated in advanced stage of gastric cancer, it may contribute to expansion, growth, invasion and metastasis of these tumor cells.

As investigated, membrane-associated serine proteases play a vital role in tumor invasion and metastasis^38^,^39^. Hepsin expression may contribute to gastric cancer progression and metastasis by a few molecular mechanisms. For instance, hepsin could act as a growth factor, which plays a role in stimulating the proliferation and increase progression ability of cancer cells^40^,^41^. This hepsin activity for cultured hepatocytes has been determined^21^. Hepsin may also directly or indirectly degrade extracellular matrix proteins by activating matrix metalloproteinases (MMPs)^42^. As well known, proteolytic digestion of extracellular matrix proteins plays a crucial role in tumor invasion and metastasis. High hepsin expression in gastric cancer tissues may at some certain reflect the state of poorly differentiated gastric cancer cells. Testing gastric cancer models in hepsin-deficient mice should indicate the biological significance and the true molecular mechanism of hepsin in gastric cancer.

In conclusion, our study has demonstrated that increased hepsin expression is correlated with poor prognosis in gastric cancer patients, and hepsin may be identified as an independent prognostic factor and may be a potential target for the treatment of gastric cancer patients. In order to better understand hepsin’s physiological functions, additional experimentation remains to be determined.

## Materials and Methods

### Cell culture and reagents

Human gastric cancer cell lines AGS and MGC80-3 were purchased from the Cell Bank of the Type Culture Collection of the Chinese Academy of Sciences (Shanghai, China), and cultured in Dulbecco’s modified Eagle’s medium (DMEM) or RPMI1640 supplemented with 10% fetal bovine serum (FBS) (catalogue no. 16000-044; Gibco, Grand Island, NY, USA) at 37 °C in a humidified atmosphere containing 5% CO_2_ separately. The gastric cancer cell lines used have been authenticated and tested to exclude the possibility of mycoplasma contamination.

### Patient samples

For tissue microarray (TMA) detection, human gastric cancer tumor specimens containing 220 cases of tumor and matched non-tumor tissues were all enrolled between 2004 and 2008 from the Department of General Surgery, Zhongshan Hospital, School of Medicine, Fudan University, Shanghai, China. Another independent set of patients comprising a total of 102 patients from Zhongshan Hospital was recruited during 2005. All the patients received standard gastrectomy with lymphadenectomy by the same surgical team. None of these patients received any preoperative anticancer therapy. The clinicopathologic features of patients are provided in Table 1. The tumor differentiation grade and clinical stages were reclassified according to the seventh American Joint Committee on Cancer (AJCC) TNM classification. The cases of gastric cancer in our study were selected only if clinical data were available. We calculated the follow-up time from the date of surgery to the date of death, or last visit. An additional independent group of 40 paired frozen gastric cancer and corresponding normal mucosa tissues were also obtained from the Department of General Surgery, Zhongshan Hospital, Fudan University, Shanghai, China, in 2014. The use of human tissue samples and clinical data was approved by the Clinical Research Ethics Committee of Zhongshan Hospital, Fudan University. All donors provided written informed consent to donate their samples. All methods were taken in accordance with the approved guidelines of School of Medical graduate Fudan University.

### Real-time PCR

Real-time PCR analyses were carried out as described previously^43^. The total RNA was isolated from the normal gastric tissues, gastric cancer tissues and matched peritumor tissues by using TRIzol reagent (Invitrogen, USA) according to the manufacturer’s instruction. Hepsin mRNA expression levels were determined by using specific primers after normalization with glyceraldehyde 3 phosphate dehydrogenase (GAPDH). The primer sequence were: GAPDH, (Forwad) 5′-GTCAAGGCTGAGAACGGGAA-3 and (Reverse) 5′-AAATGAGCCCCAGCCTTCTC-3; Hepsin, (Forward) 5′-GTCTGCAATGGCGCTGACTTCT-3′ and (Reverse) 5′-TCCGAGAGATGCTGTCCTCACA-3′.

### Western blotting

Western blot analyses were performed as described previously^44^. Primary antibodies were rabbit anti-hepsin (Abcam, UK), mouse anti-actin (Cell Signal Technology, USA). And species-specific (mouse or rabbit) secondary antibody was purchased from Santa Cruz Biotechnology.

### Immunochemistry staining

Immunohistochemical staining protocol was constructed as previous described^43^. Depending on the staining extent, the score of hepsin expression was conducted: 0, 0–5%; 1, 5–25%; 2, 26–50%; 3, 51–75%; and 4, >75%, and the staining intensity was categorized as follows: no staining scored 0, weakly staining scored 1, moderately staining scored 2 and strongly staining scored 3, respectively. The staining score was designated by multiplying staining area score by staining intensity score, yielding a series of results ranging from 0 to 12. High expression was considered as a total score >4 and low expression with a total score ≤4 according to receiver operating characteristic (ROC) analysis. The immunochemistry staining scores were determined independently by two pathologists who were blinded to the patients’ clinical data.

### Immunofluorescent staining

Immunofluorescent staining analyses were carried out as described previously^43^. Hepsin was detected with Anti-V5 (Invitrogen) and visualized with goat anti-mouse IgG-Alexa Fluor 488 (Jackson). 5 mg/ml DAPI (Beyotime) was used for nuclear staining. Images were taken by a Confocal Laser Scanning Microscope (Leica TCS SP5, Germany).

### Transwell assay

Transwell assay was performed as described previously^45^. AGS cells were transfected with plasmids, V5-hepsin and V5-hepsin N112Q. Migration and invasion of cells were determined 24 h and 40 h later, respectively. The infiltrating cells were stained with crystal violet, and cell numbers were counted from five fields. Each experiment was repeated three times.

### Statistical analysis

SPSS19.0 (SPSS Inc.; Chicago, IL, USA) and GraphPad Prism 5 (San Diego, CA) software were used for statistical analyses and graphical representations. The χ2 test was used to analyze the relations between hepsin expression and clinicopathological parameters in gastric cancer patients. Survival curves were evaluated using the Kaplan-Meier method, and differences between survival curves were tested by the log-rank test. Cox proportional hazards regression model was used to examine univariate and multivariate analyses. Only significantly different variables in univariate analysis were entered into the next multivariate analysis. Nomogram was generated by R software with “rms” package. Calibration plot for 5-year overall survival was constructed to examine the performance characteristics of the generated nomogram. The prognostic accuracy was measured by calculating the Harrell’s concordance index (c-index). A two-sided P-value < 0.05 was considered statistically significant.

## Additional Information

**How to cite this article**: Zhang, M. *et al*. High Hepsin expression predicts poor prognosis in Gastric Cancer. *Sci. Rep.* **6**, 36902; doi: 10.1038/srep36902 (2016).

**Publisher’s note**: Springer Nature remains neutral with regard to jurisdictional claims in published maps and institutional affiliations.

## Supplementary Material

Supplementary Information

## Figures and Tables

**Table 1:** Relation between intratumoral Hepsin expression and clinical characteristics of gastric cancer.

Factor	Patients		Hepsin expression		
	No.	%	Low	High	P-value
All patients	220	100	134	86	
Age (years)					0.167
≤60	110	50	72	38	
>60	110	50	62	48	
Gender					0.983
Female	74	33.6	45	29	
Male	146	66.4	89	57	
Localisation					0.637
Proximal	37	16.8	24	13	
Middle	47	21.4	26	21	
Distal	136	61.8	84	52	
Differentiation					**0.001**
Well + Moderately	41	18.6	16	25	
Poorly	179	81.4	118	61	
Lauren classification					**0.001**
Intestinal type	154	70	83	71	
Diffuse type	51	23.2	42	9	
Mixed type	15	6.8	9	6	
T classification					**0.011**
T1	40	18.2	33	7	
T2	14	6.4	10	4	
T3	65	29.5	35	30	
T4	101	45.9	56	45	
N classification					**0.039**
N0	62	28.2	45	17	
N1	40	18.2	26	14	
N2	34	15.4	15	19	
N3	84	38.2	48	36	
Distant metastasis					0.849
No	209	95	127	82	
Yes	11	5	7	4	
TNM stage					**0.007**
I	38	17.3	32	6	
II	54	24.5	32	22	
III	117	53.2	63	54	
IV	11	5	7	4	
Intravascular cancer emboli					0.588
No	158	71.8	98	60	
Yes	62	28.2	36	26	

Abbreviation: TNM = tumour node metastasis. P-value < 0.05 marked in bold font shows statistical significant.

**Table 2:** Univariate and Multivariate Cox regression analyses for overal survival of gastric cancer.

Factors	Univariate		Multivariate	
	HR (95%CI)	P-value	HR (95%CI)	P-value
Age		0.0513		
≤60	1.00 (reference)			
>60	1.456 (0.997–2.124)			
Gender		0.7384		
Female	1.00 (reference)			
Male	0.934 (0.625–1.394)			
Localisation		0.214		
Distal	1.00 (reference)			
Middle + Proximal	1.288 (0.864–1.920)			
Differentiaon		0.6104		
Well + Moderately	1.00 (reference)			
Poorly	0.883 (0.547–1.425)			
Lauren classification		0.7496		
Intestinal	1.00 (reference)			
Diffuse + Mixed	0.945 (0.621–1.409)			
T classification		**<0.001**		**0.016**
T1 + T2	1.00 (reference)		1.00 (reference)	
T3 + T4	3.445 (2.290–5.181)		3.094 (1.230–7.782)	
N classification		**<0.001**		0.645
N0	1.00 (reference)		1.00 (reference)	
N+	3.145 (2.117–4.673)		1.250 (0.484–3.226)	
Distant metastasis		**<0.001**		**<0.001**
No	1.00 (reference)		1.00 (reference)	
Yes	40.7 (11.21–147.8)		3.241 (1.698–6.185)	
TNM stage		**<0.001**		**0.011**
I + II	1.00 (reference)		1.00 (reference)	
III + IV	4.544 (3.104–6.654)		2.899 (1.280–6.566)	
Intravascular cancer emboli		**0.0024**		0.815
No	1.00 (reference)		1.00 (reference)	
Yes	1.982 (1.275–3.082)		1.049 (0.702–1.567)	
Hepsin expression		**0.0014**		**0.036**
Low	1.00 (reference)		1.00 (reference)	
High	1.913 (1.284–2.850)		1.498 (1.026–2.185)	

Abbreviation: 95% CI = 95% confidence interval; HR = hazard ratio; TNM = tumour node metastasis; P-value < 0.05 marked in bold font shows statistical significant.

**Table 3:** Comparison of the prognostic accuracies of TNM staging and Hepsin expression.

Model	C-index
Hepsin	0.5802
TNM	0.6876
TNM + Hepsin	0.7201

Abbreviations: C-index = Harrell’s concordance index.
