# Supplementary material for: High Hepsin expression predicts poor prognosis in Gastric Cancer
Source: Sci Rep. 2016 Nov 14;6:36902. doi: 10.1038/srep36902 (PMC5107942; doi:10.1038/srep36902)
Supplement: Supplementary Information [file srep36902-s1.pdf]

High Hepsin expression predicts poor prognosis in Gastric Cancer

Mingming Zhang[1], Junjie Zhao[2], Wenyi Tang[1],Yanru Wang[3],Peike Peng[1], Lili Li[1], Shushu Song[1], Hao Wu[1], Can Li[1], Caiting Yang[1], Xuefei Wang[2*], Chunyi Zhang[1*], Jianxin Gu[1]

Supplemental Figure 1

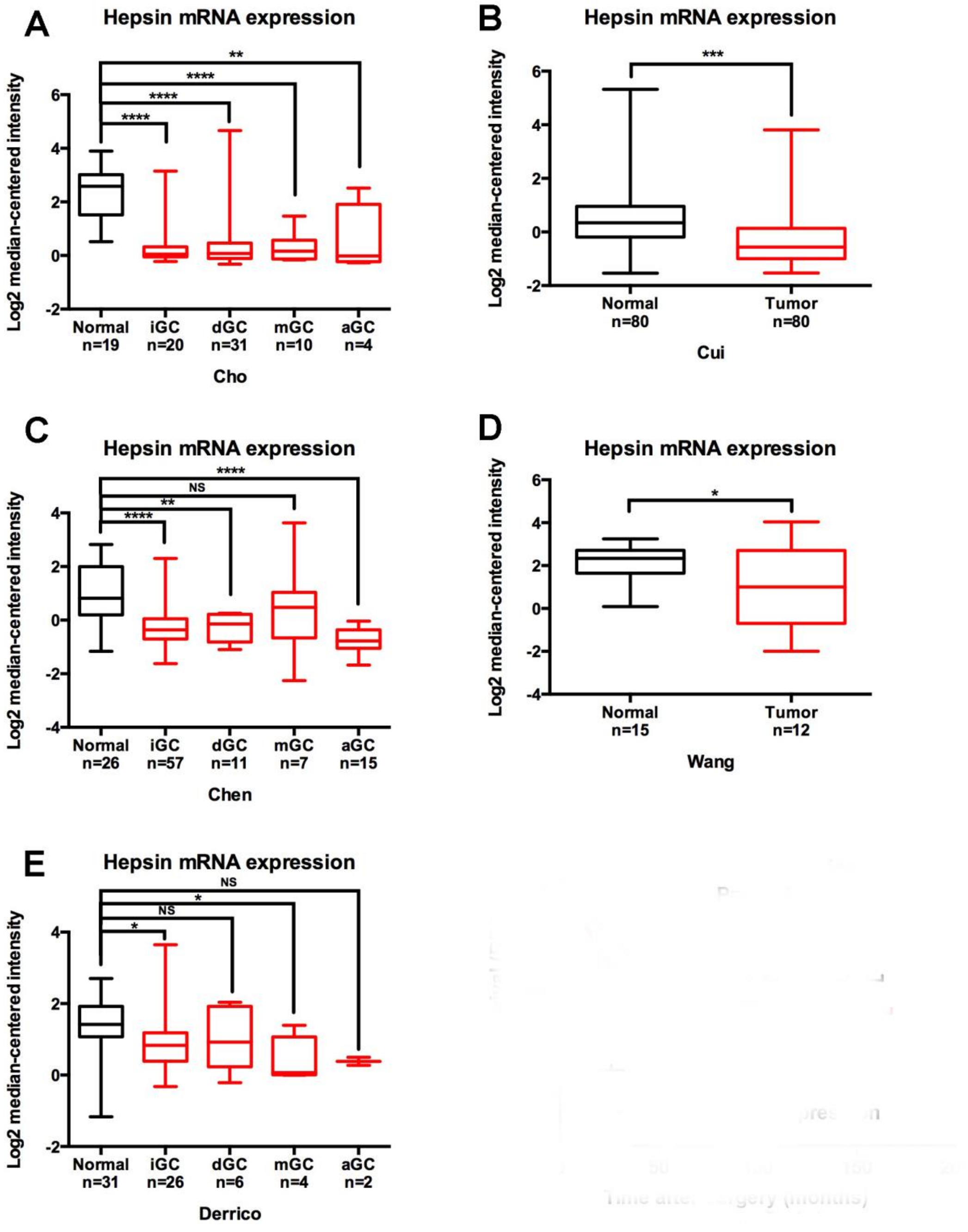

Supplementary Figure 1. The graphs for the individual datasets separately and the graphs for Normal Vs Tumors as well as across sub-types within tumors. (**A-E**)

Supplemental Figure 2

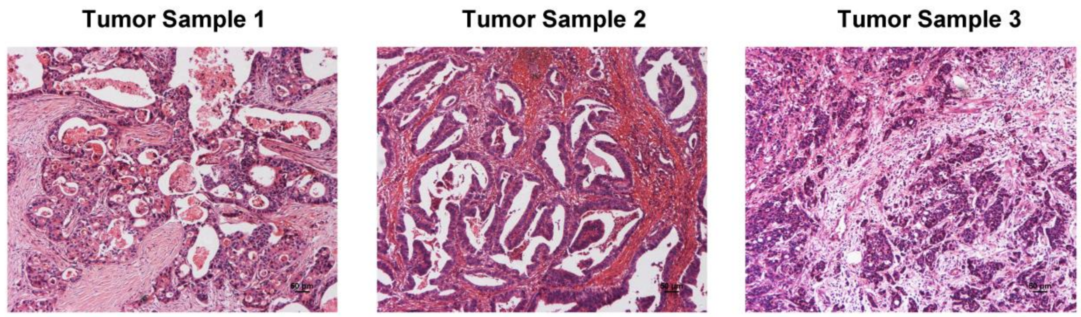

Supplementary Figure 2. Representative images of three tumor samples stained by hematoxylin-eosin (HE). The tumor samples have similar tumor cell content (60% approximately) and would be used to detect the mRNA or protein expression according to HE staining.

Supplemental Figure 3

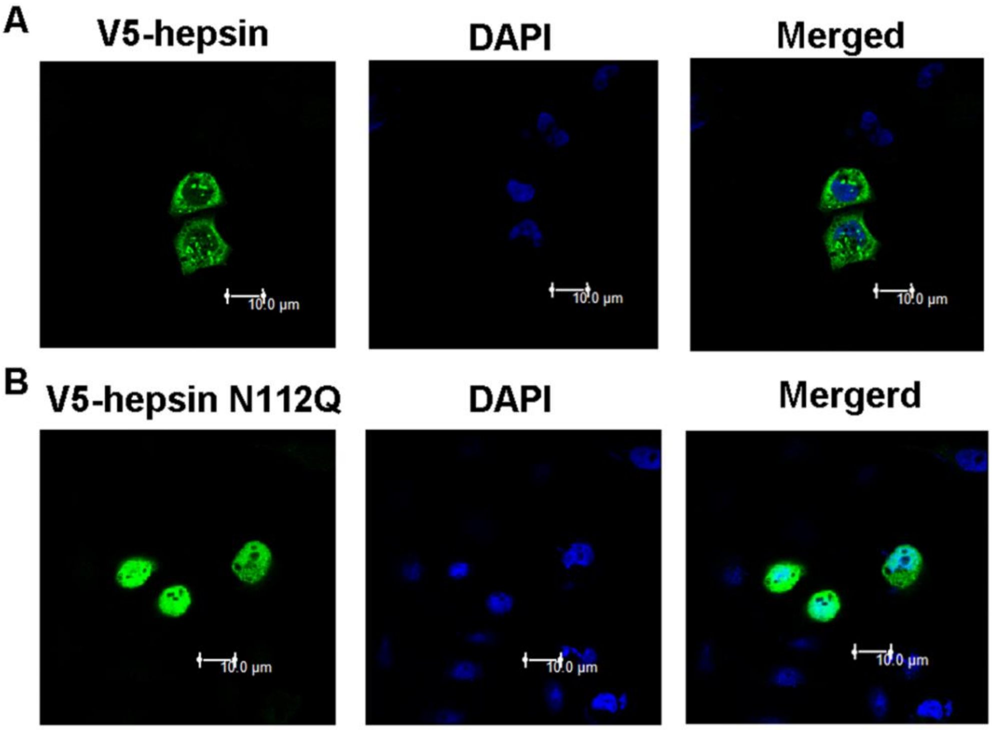

Supplemental Figure 3. Confocal microscopy revealed localization of hepsin in AGS cells. (A,B) Cells were transfected with V5-hepsin plasmid, V5-hepsin N112Q plasmid. 48 hours later, confocal immunofluorescence microscopy assay was performed. Hepsin (green) and DAPI (blue) are shown. Scale bars = 10 μm.

Supplemental Figure 4

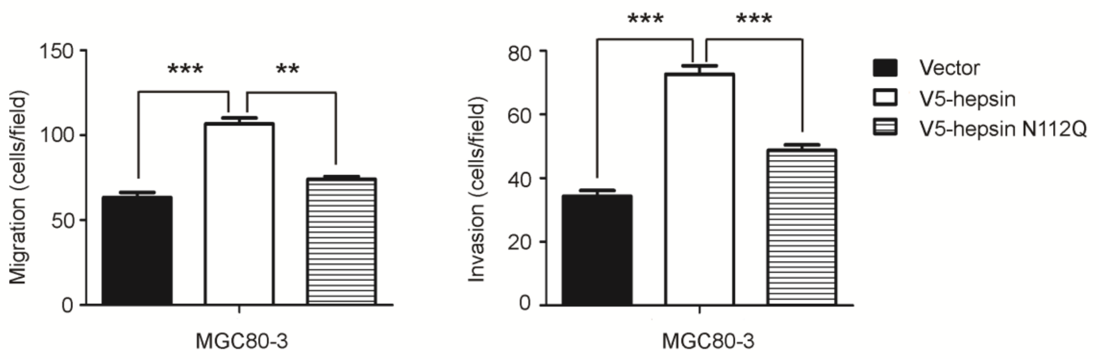

Supplemental Figure 4. The effects of wild hepsin and hepsin mutant N112Q on the migration and invasion in MGC80-3 cells were examined with a transwell assay.

Supplemental Figure 5

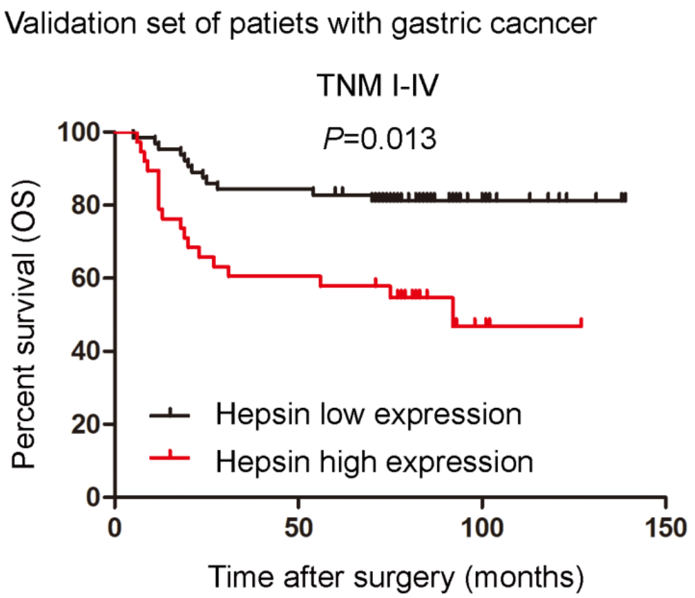

Supplementary Figure 5. Kaplan–Meier analysis for overall survival of patients with gastric cancer according to hepsin expression in validation set, n=102, P=0.013. P-value was calculated by log-rank test.

Supplemental Figure 6

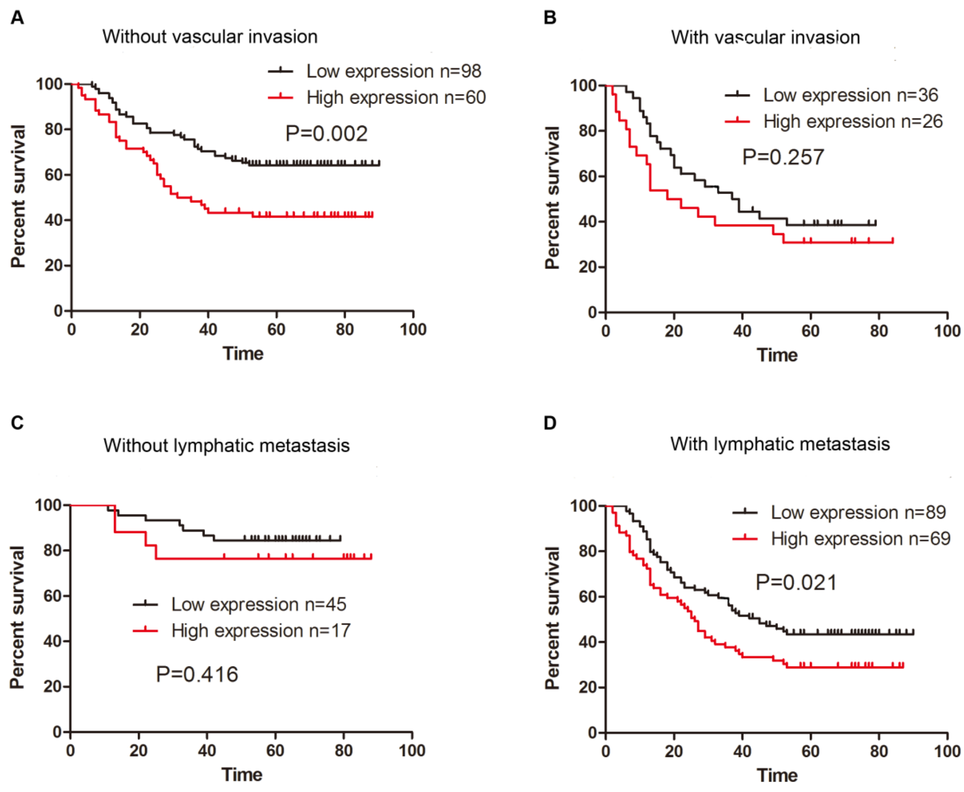

Supplemental Figure 6. High expression of hepsin was associated with poor overall survival without vascular invasion or with lymphatic metastasis. **(A,B)** Comparisons of overall survival between hepsin low expression and hepsin high expression in gastric cancer patients with or without vascular invasion. **(C,D)** Comparisons of overall survival between hepsin low expression and hepsin high expression in gastric cancer patients with or without lymph node metastasis. *P-values were calculated by log-rank test.*

Supplemental Figure 7

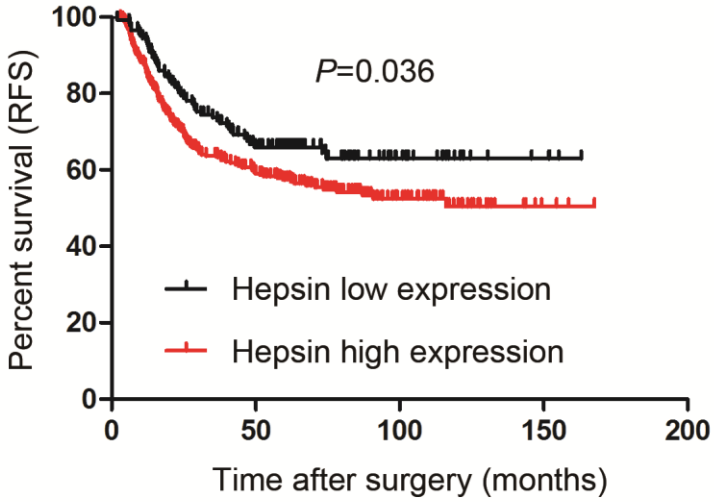

Supplemental Figure 7. The recurrence free survival (RFS) information of GSE26253.

Supplemental Table 1

Relationship between intratumoral Hepsin expression and clinical characteristics in the validation set of patients with gastric cancer					
**Factor**	**Patients**		**Hepsin expression**		
	**No.**	**%**	**Low**	**High**	**P-value**
**All patients**	102	100	64	38	
**Age (years)**					0.683
≤60	50	49	30	20	
＞60	52	51	34	18	
**Gender**					0.947
Female	38	37	24	14	
Male	64	63	40	24	
**Localisation**					0.961
Proximal	10	10	6	4	
Middle	42	41	26	16	
Distal	50	49	32	18	
**Differentiation**					0.299
Well + Moderately	41	41	23	18	
Poorly	61	59	41	20	
**Lauren classification**					**0.038**
Intestinal type	45	44	22	23	
Diffuse type	27	26	20	7	
Mixed type	30	30	22	8	
**T classification**					**0.033**
T1	31	30	25	6	
T2	10	10	7	3	
T3	4	4	3	1	
T4	57	56	29	28	
**N classification**					**0.031**
N0	43	42	31	12	
N1	19	19	13	6	
N2	14	14	10	4	
N3	26	25	10	16	
**Distant metastasis**					0.554
No	99	97	63	36	
Yes	3	3	1	2	
**TNM stage**					**0.023**

I	35	34	26	9	
II	19	19	15	4	
III	45	44	22	23	
IV	3	3	1	2	
Abbreviation: TNM=tumour node metastasis. P-value＜0.05 marked in bold font shows statistical significant.					
